# Androgen Plays a Potential Novel Hormonal Therapeutic Role in Th17 Cells Predominant Neutrophilic Severe Asthma by Attenuating BECs Regulated Th17 Cells Differentiation via MBD2 Expression

**DOI:** 10.1155/2022/3096528

**Published:** 2022-08-25

**Authors:** Binaya Wasti, Zhifeng Chen, Yu Yuan, Yi He, Wentao Duan, Jingsi Jia, Danhong Li, Bing Xiao, Jianmin Li, Yi Liu, Shaokun Liu, Dongshan Zhang, Xiufeng Zhang, Libing Ma, Xudong Xiang

**Affiliations:** ^1^Department of Respiratory Medicine, Hunan Centre for Evidence-Based Medicine, Research Unit of Respiratory Diseases, The Second Xiangya Hospital of Central South University, 139 Middle Renmin Road, Changsha, Hunan 410011, China; ^2^Department of Emergency, The Second Xiangya Hospital of Central South University, 139 Middle Renmin Road, Changsha, Hunan 410011, China; ^3^Department of Respiratory and Critical Care Medicine, Hunan Provincial People's Hospital, Changsha, China; ^4^Department of Respiratory Medicine, Zhuzhou City Central Hospital, Zhuzhou, China; ^5^Department of Respiratory Medicine, The Second Affiliated Hospital of Hainan Medical University, Haikou, China; ^6^Department of Respiratory and Critical Care Medicine, The Affiliated Hospital of Guilin Medical University, Guilin, China

## Abstract

T helper 17 (Th17) cells subtype of non-T2 asthma is less responsive (resistant) to inhaled corticosteroids (ICS), so also called severe asthma. Methyl-CpG-binding domain protein 2 (MBD2) regulates the differentiation of the Th17 cells, showing the possibility of a therapeutic target in severe asthma. Androgen tends to show beneficial therapeutic effects and is a “hot research topic,” but its role in the differentiation and expression of Th17 cells via MBD2 is still unknown. The aim of this study was to evaluate how sex hormone interacts with MBD2 and affects the differentiation and expression of Th17 cells in severe asthma. Here, first, we measured the concentration of androgen, estrogen, and androgen estrogen ratio from subjects and correlated it with severe asthma status. Then, we established an animal model and bronchial epithelial cells (BECs) model of severe asthma to evaluate the role of MBD2 in the differentiation and expression of Th17 cells (IL-17), the therapeutic potential of sex hormones in severe asthma, and the effect of sex hormones in BECs regulated Th17 cells differentiation via MBD2 at the cellular level. Increased MBD2 expression and Th17 cells differentiation were noted in the animal and the BECs severe asthma models. Th17 cell differentiation and expression were MBD2 dependent. Androgen attenuated the differentiation of BECs regulated Th17 cells via MBD2 showing BECs as a therapeutic target of androgen, and these findings postulate the novel role of androgen in Th17 cells predominant neutrophilic severe asthma therapy through targeting MBD2.

## 1. Introduction

Asthma is a heterogeneous disease with different phenotypes (clinical presentations) and endotypes (pathobiological mechanisms). The non-T2 asthma (T2 low), sometimes also known as noneosinophilic asthma, is still less well-characterized and is mainly of late-onset neutrophilic, pauci-granulocytic, or mixed types, whereby inflammation is usually driven by varieties of cells and cytokines. It may be associated with muscular or neural dysfunction as well as comorbidities like gastroesophageal reflux disease and obesity [1, 2]. Non-T2 asthma is generally resistant or less sensitive to inhaled corticosteroids (ICS), hence also known as severe asthma.

Th17 cell is entitled as the kernel of severe asthma, as it is the main influencing factor in the pathogenesis of severe asthma as evidenced by studies [3–7]. Briefly, Th17 cells differentiation starts with T cell receptor stimulation, plus accessory factors activate the expression of signal transducer and activator of transcription 3 (STAT3), which in term facilitates the expression of retinoic acid receptor-related orphan nuclear receptor gamma t (ROR*γ*t), a master regulator for the production and differentiation of Th17 cells [8]. Transforming growth factor *β* (TGF-*β*), mainly responsible for bronchial hyperreactivity and the structural changes of the bronchial wall in asthma and other various conditions, is produced by the activated Treg cells, and Treg cells also promote Th17 cells differentiation via TGF-*β*, and transfer of Treg cells enhanced Th17 cells (IL-17) production is associated with the systemic autoimmune disease in the animal model [9]. The undesired expansion of Th17 cells might disrupt Treg cells, and the disruption of Th17/Treg cells homeostasis is associated with asthma severity and exacerbation [10]. Collectively, the interaction of Th17 cells (IL-17), STAT3, ROR*γ*t, TGF-*β*, and Treg cells is complex making mysterious asthma puzzle. However, out of all, we will only explore Th17 (IL-17) cells and ROR*γ*t under various conditions in this study.

Neuronal and hormonal responses, oxidative stress, inflammatory cytokines, and epigenetics mechanism are involved in the pathogenesis of Th17 cells and mediate neutrophils predominant severe asthma. However, the underlying pathogenesis mechanisms are still not clear.

Methyl-CpG-binding domain protein 2 (MBD2) is a critical player in asthma epigenetics and immunity that determines the transcriptional state of the epigenome, interprets DNA methylation marks, induces posttranscriptional histone modification, and regulates the target genes [11, 12]. MBD2 is involved in the differentiation of the Th17 cells in the pathogenesis of severe asthma where its overexpression increases the Th17 cells (IL17 protein) and silencing results in decreased expression as shown from our previous studies [13, 14]. Its deficiency inclines toward Th2 cellular polarization [15]. Our recent study also showed that MBD2 increases significantly in severe asthma and is a potential biomarker for identifying severe asthma various endotypes [16].

Sex hormones are not only involved in sex differentiation and reproduction, but are also involved in the regulation of immune response, and signaling pathways have become a “hot research topic” in asthma [17–19]. Estrogen and progesterone promote the differentiation of the Th17 cells, IL-17A-mediated airway inflammation, and IL23R expression through inhibiting let-7f microRNA expression [20, 21]. Androgens negatively regulate ILC2, and Th2 cells, as well as reduce allergic inflammation in males [22, 23]. Also, TES diminishes the IL-17A protein expression reducing neutrophilic airway inflammation [23, 24]. Moreover, asthma is more prevalent in men than women with aging as the androgen level decreases [25, 26] and aggravation of asthma symptoms noted during premenstrual and the menstrual phase seems to be correlated with reduced testosterone levels [27]. Does it mean androgen administration is beneficial in asthma?

At present, we do not know the role of sex hormones especially in MBD2-mediated Th17 cells predominant neutrophilic severe asthma. Does sex hormone down-regulate MBD2 expression or directly affects the differentiation and the expression of the Th17 cells in severe asthma as a therapeutic potential? In our recent review, we evaluated the correlative association of sex hormones fluctuation status, MBD2 expression, and Th17 cells, and postulated androgenic therapeutic potential in Th17 cells predominant neutrophilic severe asthma via regulating MBD2 [19], and here performed this study. First, we measured the androgen, the estrogen, and the androgen estrogen ratio in severe asthma subjects and correlated them with asthma status. Second, severe asthma animal model and the BECs cellular model were established to evaluate the expression, influence, and role of MBD2 on Th17 cells, comparison of male and female sex hormone therapeutic efficacy in severe asthma, and sex hormones affecting the expression of Th17 cells via regulating MBD2 in BECs as a cellular level working mechanism of sex hormone in severe asthma.

Here, we evaluate whether sex hormones (androgen) can offer a novel potential therapeutic role in the treatment of Th17 cells predominant neutrophilic severe asthma through regulating MBD2 expression.

## 2. Materials and Methods

### 2.1. Subjects

All voluntary subjects (normal control, 20; asthma, 31; and severe asthma, 32), ex-smoker with less than 10 pack-years or never smokers, were recruited from 01 November 2021 to 15 April 2022 at Second Xiangya Hospital of Central South University, after written informed consent and ethical approval. Asthmatic subjects were diagnosed according to GINA guidelines [28], and normal controls were without any obvious symptoms, atopy, and chronic diseases. Asthma control was accessed with an asthma control test (ACT) [29] score with 20-25 as well-controlled and below 20 poorly controlled, and severity was assessed by pulmonary function test (PFT), ACT, and medication use as mild, moderate, and severe [30, 31]. Mild and moderate asthma were labeled as “asthma” to separate from severe asthma. Subjects associated with acute asthma, cardiac asthma, ABPA, bronchogenic carcinoma, complicated solid tumor/malignancies, hematologic/autoimmune disorders, pregnancy/lactation, and having serious infections were excluded from the study.

### 2.2. Baseline Evaluation

The demographic information of each subject was recorded. Blood samples were examined for neutrophil, eosinophil, and IgE levels. Spirometry was performed to evaluate the lung function, and each parameter was evaluated and compared with each group.

### 2.3. ELISA for DHT (Dihydrotestosterone, an Androgen), E2 (17*β*-Estradiol, an Estrogen), and DHT: E2 Ratio Measurement

Blood samples from subjects were examined for ELISA to measure the DHT, E2, and DHT: E2 ratio as instruction provided by the manufacturer (APExBIO, USA). From here onwards, DHT as an androgen and E2 as an estrogen have been used frequently and interchangeably in the manuscript.

### 2.4. Mice and Ethical Approval

Ethical Review Committee of Xiangya Second Hospital, Central South University, approved the animal experiment, and the study was conducted in compliance with animal care and use committee guidelines. 6-7 weeks (approx. wt 20-22gms), C57BL/6 female mice provided from animal center kept in an SPF environment with 12/12 h light/dark cycle for the experiments.

### 2.5. Experimental Reagents and Equipment

GREER laboratories supplied HDM; OVA, LPS, and aluminum hydroxide gel from Sigma; mice spirometer (MAX 1320) from Buxco®, USA; Beijing Zhongshidichuang Science and Technology Development Co. Ltd. supplied animal asthma inducing instrument (YLS-8A); centrifuge (5810 R) from Eppendorf®, Germany; image analyzer (Leica Application Suite V4) from Leica, Germany; and Olympus, Japan (Leica®) supplied optical microscope (DMI3000B); 150-mesh cell sieve (Biosharp, BS-100-XBS, China); bronchial epithelial growth medium (Procell, CM-M007, China); DAPI and cytokeratin specific monoclonal antibody (pan-Cytokeratin, Santa Cruz, sc-8018, USA); leukocyte activation cocktail (550583, BD Biosciences, USA); cell viability marker (Fixable Viability Stain 510 antibody, BD Pharmingen); PE-anti-IL-4 (BD Pharmingen); APC-anti-IL-17A (Biolegend); Lipofectamine 3000 (Invitrogen, USA); bicinchoninic acid (BCA), Beyotime, Shanghai, China; DHT (B8214) and 17*β*-estradiol (C4348) APExBIO, USA; MBD2 antibody (Abcam, Cambridge, USA); eosinophil antibody (anti-ECP, Biorbyt, Cambridge, UK); neutrophil antibody (anti-Gr-1, Biolegend, San Diego, USA); GATA3 (10417-1-AP) and *β* actin (60008-1-Ig) Proteintech, USA; ROR*γ*t (EPR20006) abcam, USA.

### 2.6. Mice Asthma Model

Mice were divided into normal control, asthma (conventional asthma), severe asthma, severe asthma+ DHT, severe asthma+ E2, and severe asthma+ DHT/E2 groups. Each group contained at least 6 mice. The asthma model was followed and established as reported [32]. Severe asthma, severe asthma+ DHT, severe asthma +E2, and severe asthma+ DHT/E2 mice were sensitized intraperitoneally on days 0, 1, and 2 with 100 *μ*g HDM (house dust mites) +100 *μ*g OVA (ovalbumin) +15 *μ*g LPS (lipopolysaccharides) +2 mg aluminum hydroxide (Al(OH)3), and OVA atomized 30 minutes before intranasal HDM excitation on days 14, 15, 18, 19, and 20. The asthma group received 25 *μ*g OVA +1 mg Al(OH)3 intraperitoneally on the 0 and 7th days, and then, OVA atomized for 30 minutes on the 14th to 20th days [33]. Saline was only injected in the normal control group for sensitization and atomization on the same days, site, and dose as the severe asthma group. On day 21, mice were sacrificed.

### 2.7. Injection of Androgen and Estrogen

Subcutaneous depot injection of 3.6 *μ*g/g body wt. of DHT (the most bioactive metabolite of testosterone) [34] dissolved in sesame oil was injected in severe asthma+ DHT group of mice, 2.5 *μ*g/mouse of E2 [35] subcutaneous depot injection in sesame oil was given to severe asthma+ E2 group, and 3.6 *μ*g/mouse DHT:2.5 *μ*g of E2 was injected in severe asthma+ DHT/E2 group of each mouse as indicated above on days 11 to 20.

### 2.8. Airway Hyperresponsiveness

On day 21 (24 h after final challenge), baseline (RL0), and methacholine (Mch) induced airway hyperresponsiveness measured by Buxco Electronics, RC System, USA, plethysmograph as previously described [36] in tracheotomized intubated mice after anesthesia. 10 *μ*l Mch mixed with 10 *μ*l saline used for initiation of broncho-provocation, and Mch increased to 0.39 mg/ml (dose 1), 0.78 mg/ml (dose 2), 1.56 mg/ml (dose3), and 3.12 mg/ml (dose 4), and change in lung resistance (RLX) recorded for analysis.

### 2.9. BALF (Broncho-Alveolar Lavage Fluids) Cell Count

BALF was collected as described previously [36]. Hematocytometer determined total BALF cell counts after the removal of red blood cells by centrifuge, and precipitation. After cell slicing and H&E staining, BALF NEU (neutrophils) and EOS (eosinophils) were determined in 200 total BALF cells.

### 2.10. Histopathological Analysis and Immunohistochemistry

After 10% formalin fixation via the tracheal route, the lungs were stored in 10% formalin. Fixed lung tissues were paraffinized and sectioned (5 *μ*m) for hematoxylin and eosin (H&E stain, 2-4 sections per group), immunohistochemistry for the protein expression carried out by specific antibodies for NEU (anti-Gr1), EOS (anti-ECP), and MBD2.

### 2.11. Bronchial Epithelial Cells (BECs) Isolation and Culture

BECs were isolated as described [37, 38]. Briefly, the dissected airways were transferred to a minimum essential medium (Fisher Scientific International, MEM, 11095-080) after PBS washing and preheated with Roche Diagnostics 0.1 mg/ml DNase and 1.4 mg/ml pronase to 37°C. One hour later, epithelial cells from the airways were separated by inverting the tube 12 times, and 1 ml of fetal bovine serum from FBS, Gibco, Australia, was added to stop enzyme digestion. Undigested excess tissues were removed by a 150-mesh cell sieve. After 5 minutes of centrifugation at 800×*g* and supernatant discard, cells were resuspended with MEM containing 10% FBS. Then, cells were inoculated in culture bottles at 37°C for 2 hours with 5% CO2 to remove contaminated nonepithelial cells. The suspended cells with culture medium were collected and centrifuged at 800×*g* for 5 min to remove the supernatant. Finally, BECs were cultured with a bronchial epithelial growth medium at 37°C with 5% CO2 in a humidified incubator.

Cytokeratin is an epithelial marker, and about 95% of epithelial cells are positive for it and are not expressed by lymphocytes [39]. BECs were centrifuged and stained with cytokeratin-specific monoclonal antibody and DAPI.

### 2.12. BECs Asthma Model and Transfection

Th17 cells predominant cellular severe asthma model was induced by treating BECs with 100 *μ*g/ml HDM and 100 ng/ml LPS, 100 *μ*g/mL HDM to induce asthma (T2 asthma) and PBS for normal controls [40]. Small interfering RNA targeting MBD2 (siR-MBD2) and negative control (siR-NC) were purchased from RiboBo (Guangzhou, China). The MBD2 interfering sequence was 5′-GCAAGATGATGCCTAGTAA-3′. Mouse MBD2, OE-MBD2 plasmids, and negative control (OE-NC) were purchased from HonorGene (Hunan, China). Small interfering MBD2 RNA and plasmid transfected in BECs for 48 h using Lipofectamine 3000. Finally, 2 days later, cells were treated with 100ug/ml HDM and 100 ng/ml LPS for 24 h.

### 2.13. DHT, E2, and DHT/E2 Pretreatment with BECs, CD4+ T Cell Isolation, and Cocultivation with BECs

Magnetic bead separation (130–049-201, Miltenyi Biotec, Germany) isolated mice splenic CD4+ T cells. The asthma model transfected BECs were pretreated with 10 nM of DHT [41], 1 nM of E2 [42], and 10 : 1 nM of DHT:E2 (see subsection 4.2.3) in severe asthma + DHT, severe asthma +E2, and severe asthma +DHT/E2 groups, respectively, for 24 h, cocultured with CD4+ T cells at a ratio of 10 : 1 (TCs: BECs) for 24 h in complete RPMI 1640 culture medium (Gibco, Australia) supplemented with 10% FBS, 1% penicillin and streptomycin, soluble anti-CD28 (1.0 *μ*g/ml, eBioscience), soluble anti-cd3e (0.5 *μ*g/ml, eBioscience), and IL-2 (20 ng/ml, eBioscience). To analyze T cell subsets, cells were collected after 24 h, and flow cytometry determined the concentration of IL-4 and IL-17A to obtain the ratio of Th2 to Th17 cells. BECs MBD2 was extracted by western blotting.

### 2.14. Flow Cytometry

Leukocyte activation cocktail (2 *μ*l/ml) stimulated the CD4+ T cells (1 × 106 cells/ml), cultured for 6 hours in 5% CO2 at 37°C, and finally collected for cytometry analysis. Then, cells were stained with cell viability marker for 15 mins at room temperature in dark after 6 hours of incubation. The cells were stained with FITC-anti-CD4 antibody (Biolegend) for surface markers, fixation, and permeabilization performed by Cytofix/Cytoperm Soln Kit (BD Pharmingen) in dark at 4°C for half an hour, and then washed with permeabilization buffer and stained with PE-anti-IL-4 and APC-anti-IL-17A antibodies in permeabilization buffer at 4°C in the dark for half an hour. Finally, flow cytometry was performed, and data were analyzed using FACS Canto II (Becton Dickinson) and FlowJo version X software.

### 2.15. Western Blotting

Lungs were crushed and lysed in radioimmunoprecipitation (RIPA) lysis buffer added with 1% protease inhibitors (Beyotime, Shanghai, China) to prepare proteins. Proteins in the cellular asthma model were also prepared in the same buffer. We measured the protein concentration by bicinchoninic acid (BCA) assay according to the manufacturer's instructions (Beyotime, Shanghai, China). We transferred the 30 *μ*g of protein to the membrane after 1-1.5 h of sodium salt dodecyl sulfate-polyacrylamide gel electrophoresis (SDS-PAGE), and Tris-buffered saline-Tween 20 (TBST) is used to wash the membrane for 5 mins and then finally sealed by 5% skim milk powder for 1.5 h at room temperature. After that, the membrane was incubated with appropriately diluted MBD2, *β*-actin antibodies at 4°C overnight. The membrane was incubated with a secondary antibody at room temperature for 1 h. We obtained images with a chemiluminescence gel imaging system and Image J software (National Institutes of Health) and measured the band intensities. The relative protein expression level was calculated by the ratio of the gray value of target bands to that of *β*-actin.

### 2.16. Statistical Analysis

Experiments were performed at least 3 times, and data were expressed as mean ± standard deviation (M ± SD). Group differences were analyzed by chi-square test, Kruskal-Wallis test or one-way analysis of variance (ANOVA) followed by Dunn's multiple comparisons test. All analysis and graph generation were performed using GraphPad Prism 8.0.1 software (GraphPad Software Inc.). A *P* value <0.05 indicated a statistically significant difference.

## 3. Results

### 3.1. Subject Characteristics

The demographic characteristics, biochemical indexes, and lung function indexes of the 77 participants, including 29 with severe asthma, 29 asthma, and 19 normal control (healthy control in Table 1), were shown. Significant differences in age, sex, and BMI were noted in the groups. Subjects with severe asthma had increased neutrophils, lower FEV1, FEV1/FVC%, and FEV1%predicted (Table 1). Interestingly, IgE and eosinophils levels were higher in the asthma group and much higher in the severe asthma group compared with the HC group because the severe asthma group also contains T2 severe asthma endotypes that predominantly have IgE and eosinophils as common biomarkers as shown from our previous study [16], and T2 and non-T2 asthma may also show mixed overlapping profiles in some cases [19].

#### 3.1.1. Severe Asthma Was Associated with Higher Estrogen and Reduced Androgen Levels in Subjects

**Figure 1:**
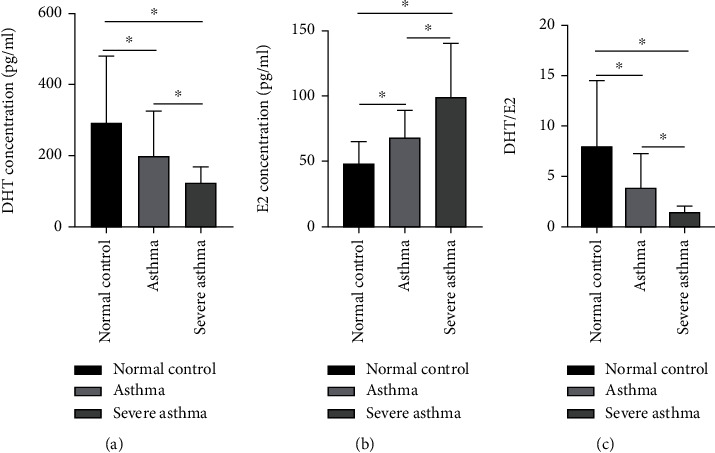
Severe asthma was associated with higher estrogen, and reduced androgen levels showing sex hormones fluctuation status correlates with asthma prevalence and remission. Severe asthma is mediated by Th17 cells, and estrogen is involved in the differentiation of Th17 cells along with its cytokines, whereas androgen reduces allergic inflammation and IL-17A protein expression. To know the concentration of sex hormones in severe asthma, we collected blood samples from different asthma groups and processed them for flow enzyme-linked immunosorbent assay (ELISA) to measure DHT, E2, and DHT/E2 ratio. Subjects were diagnosed according to GINA guidelines and mild and moderate asthma were labeled as “asthma” to separate from severe asthma. (a) Measurement of DHT. (b) Measurement of E2. (c) Measurement of DHT/E2 ratio. DHT-dihydrotestosterone as androgen, E2-17*β*-estradiol as estrogen, and DHT/E2-androgen estrogen ratio. From here onwards (in this section), DHT as androgen and E2 as estrogen have been used frequently and interchangeably. (^∗^*p* < 0.05).

Estrogen is involved in the differentiation of Th17 cells (IL-17), whereas androgen reduces allergic inflammation and IL-17A protein expression. We collect blood samples from different asthma groups to measure the concentration of DHT (an androgen), E2 (17*β*-estradiol as estrogen), and DHT: E2 by ELISA which has been shown in Figure 1. We noted that severe asthma was associated with significantly decreased DHT and higher E2 level (Figure 1).

### 3.2. Establishment of Severe Asthma Model and Effect and Concentration of DHT and E2 in the Model

**Figure 2:**
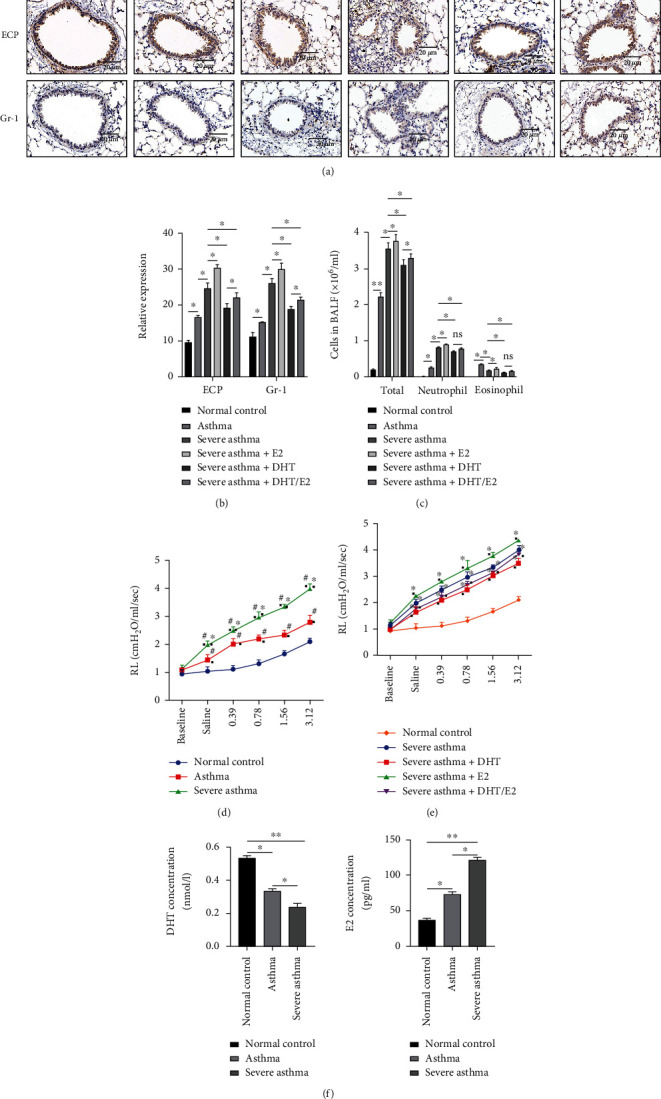
Establishment of severe asthma model, and the effect and concentration of DHT, E2, and DHT/E2 in the model. Mice were divided into normal control, asthma, severe asthma, severe asthma+ DHT, severe asthma+ E2, and severe asthma+ DHT/E2 groups. After sensitization, atomization, and excitation according to the protocol and subcutaneous injection of DHT, E2, and DHT/E2 in allocated groups, mice were sacrificed for various studies, and finally lung, and spleen harvested and serum, BALF (broncho-alveolar lavage fluid) collected. (a) Lung tissue sections were stained with hematoxylin and eosin stain (H&E), immunohistochemistry eosinophil antibody (anti-ECP), and neutrophil-specific antibody (anti-Gr-1). As estrogen is involved in the differentiation of Th17 cells and its cytokines in neutrophilic severe asthma, histological lung analysis of severe asthma+E2 group and severe asthma group showed noticeably enhanced peri-bronchial inflammation with infiltration of neutrophils (NEU), whereas severe asthma+ DHT group showed the opposite results. (b) Lung relative expression of eosinophils (EOS) and NEU protein in different groups. (c) BALF total cells, NEU, and EOS in all groups. (d) Pulmonary resistance (RL) in normal control, asthma, and severe asthma group of mice. The severe asthma group showed higher RL than the other two groups. (e) Comparison of RL in all groups including the effect of DHT, E2, and DHT/E2. The severe asthma + DHT group showed a reduced level of RL than all the groups except the normal control. (f) The concentration of DHT and E2 in normal control, asthma, and severe asthma group of mice was measured by ELISA in serum samples. Severe asthma was associated with reduced DHT and higher E2 levels showing possible involvement of estrogen in severe asthma mechanism, while the DHT as a potential therapeutic agent reversed (reduced) the effect of severe asthma. (d) #Comparison with normal control and ^∗^ comparison with asthma. (e) ^∗^ Comparison with severe asthma+ DHT and normal control group. Scale bar, 20 *μ*m. (^∗^*p* < 0.05, ^∗∗^*p* < 0.01, #*p* < 0.05; ns: not significant).

The severe asthma group of mice showed early sleepiness than the asthma group and much more early sleepiness than the normal control. The histological lung analysis of the severe asthma group showed noticeable peri-bronchial inflammation with infiltration of neutrophils, and as expected, severe asthma+E2 group showed markedly enhanced inflammation with neutrophil infiltration, while in contrast DHT, in the severe asthma+ DHT group reduced the inflammation and infiltration of neutrophils (Figure 2(a)). We also measured the relative expression of EOS and NEU protein (ECP, Gr1) and were the same as the histological analysis (Figure 2(b)). The total BALF cells and NEU count were high in severe asthma, while the severe asthma+E2 showed the highest level of total and NEU in BALF, but we observed the highest EOS count in the asthma group (Figure 2(c)). DHT in severe asthma + DHT group reduced BALF total cells and NEU. Mch inhalation increased the pulmonary resistance (RL) in severe asthma in the asthma group (Figure 2(d)). Interestingly, DHT in severe asthma + DHT reduced the RL, and RL was lower in the severe asthma+E2 and severe asthma + DHT/E2 groups (Figure 2(e)). We measure the concentration of DHT and E2 by ELISA and show a similar pattern as shown in Figure 1 (Figure 2(f)).

### 3.3. Severe Asthma Was Mediated by Th17 cells (IL-17) and the Differentiation and Expression of Th2/Th17 cells (IL-4/IL-17) under the Influence of DHT, E2, and DHT/E2

**Figure 3:**
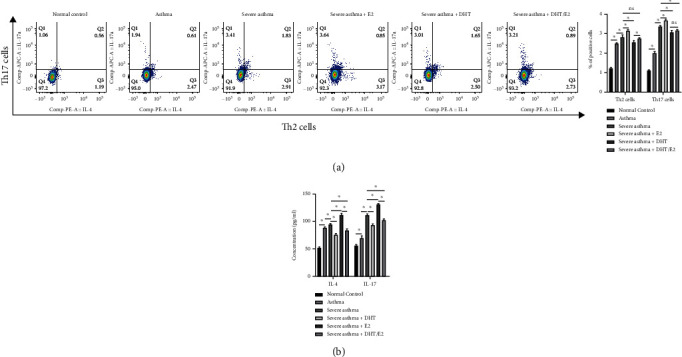
Severe asthma was mediated by Th17 cells (IL-17) and the differentiation and expression of Th2/Th17 cells (IL-4/IL-17) under the influence of DHT, E2, and DHT/E2. (a) Detection and measurement of Th2 and Th17 cells by flow cytometry from the splenocytes. The severe asthma group showed an increase in Th17 cells than asthma and the normal control group, whereas the severe asthma+E2 group showed a much more obvious increase in Th17 cells than the severe asthma+ DHT and severe asthma+ DHT/E2 groups. The percentage of Th2 and Th17 cells was also shown, and the results correlated with flow cytometric analysis. (b) Concentration of IL-4 and IL-17 in different groups from splenocytes by ELISA. Higher IL-17 concentration was observed in the severe asthma group than in asthma and normal control. Severe asthma+E2 showed an obvious increase in IL-17, while the severe asthma+ DHT significantly reduced the IL-17 showing the therapeutic potential. (^∗^*p* < 0.05; ns: not significant).

We observed abundant Th17 cells in severe asthma from splenocytes by flow cytometry analysis, and Th2 cells were mainly involved in asthma (Figure 3(a)). As we know, estrogen is involved in the differentiation of Th17 cells and their cytokines, and the addition of E2 (severe asthma+E2) increased the Th17 cells. In contrast, the DHT in severe asthma+ DHT reduced the Th17 cells more than in severe asthma, severe asthma+E2, and severe asthma+ DHT/E2 groups (Figure 3(a)). The percentages of Th2 and Th17 cells were also measured, and the results correlated with flow cytometric analysis. IL-4 is the main representative cell factor for Th2 cells, and IL-17 is for Th17. The ELISA measurement of the concentration of IL-4 and IL-17 in different groups from splenocytes showed an increased level of IL-17 in severe asthma, much increased in severe asthma+E2, while DHT in severe asthma+ DHT reduced the level as compared to severe asthma, severe asthma+E2, and severe asthma+ DHT/E2 groups [Figure 3(b)].

### 3.4. Detection of BECs, measurement of IL-4 and IL-17 from BECs supernatant, and differentiation of Th2 and Th17 cells from BECs T cell coculture under DHT, E2, and DHT/E2 influence

**Figure 4:**
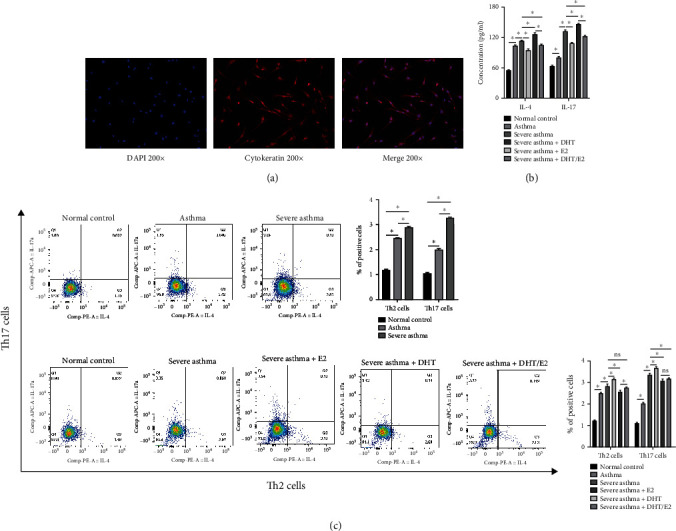
Detection of bronchial epithelial cells (BECs), measurement of IL-4 and IL-17 from BECs supernatant, and differentiation of Th2 and Th17 cells from BECs T cell coculture under DHT, E2, and DHT/E2 influence. (a) Immunofluorescence detection of a positive rate of cytokeratin, an epithelial marker, in BECs. (b) ELISA measurement of IL-4 and IL-17 from BECs supernatant. A higher level of IL-17 in severe asthma was observed than in asthma and the normal control group. The severe asthma+E2 group showed increased IL-17 expression possibly as a causative agent in severe asthma while DHT in severe asthma+ DHT reduced the IL-17 expression than severe asthma, severe asthma + E2, and severe asthma + DHT/E2 group with statistical significance. (c) After the detection of BECs from (a), we stimulated BECs with 100 *μ*g/ml house dust mite (HDM) as asthma, 100 *μ*g/ml HDM+100 ng/ml lipopolysaccharide (LPS) as severe asthma, or saline as a normal control for 24 h, DHT, E2, and DHT/E2 added in a designated group for 24 h, cocultured with CD4+ T cells for 24 h, and finally Th2 and Th17 cells were detected by flow cytometry. Percentage of Th2 and Th17 cells is also shown from different groups. (^∗^*p* < 0.05; ns: not significant).

Cytokeratin is an epithelial marker, and about 95% of epithelial cells are positive for it. The immunofluorescence detection of positive rate of cytokeratin in BECs is shown in Figure 4(a). ELISA measurement of IL-4 and IL-17 from BECs supernatant showed a higher concentration of IL-17 in severe asthma, much higher with severe asthma+E2 group, and the addition of DHT (severe asthma+ DHT) lowered the IL-17 concentration in severe asthma with statistical significance (Figure 4(b)). The flow cytometry analysis of Th2 and Th17 cells from BECs T cell coculture showed Th2 cells as a predominant marker in asthma while Th17 cells are predominantly higher in severe asthma. E2 addition in the severe asthma+E2 group increased the Th17 cells and DHT in severe asthma+ DHT reduced the same, while the DHT/E2 (severe asthma+ DHT/E2) reduced the Th17 cells, but not to the level of severe asthma+ DHT group (Figure 4(c)).

### 3.5. Detection and expression of GATA3 and ROR*γ*t in severe asthma and influence of DHT, E2, and DHT/E2 on GATA3 and ROR*γ*t expression

**Figure 5:**
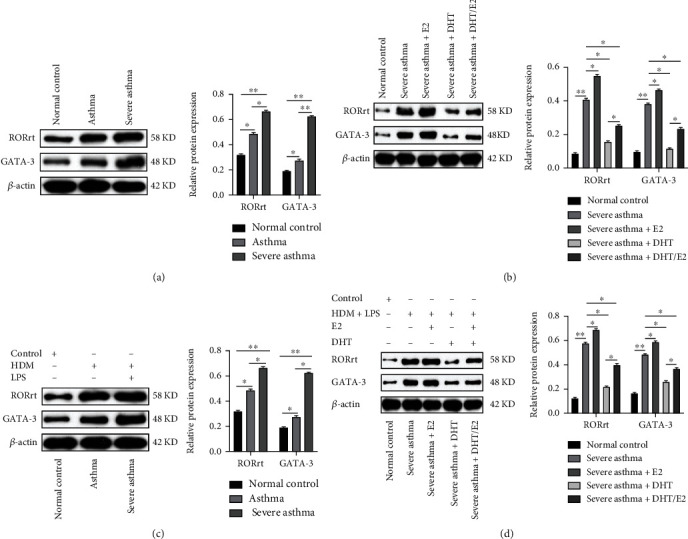
Detection and expression of GATA3 and ROR*γ*t in severe asthma and influence of DHT, E2, and DHT/E2 on GATA3 and ROR*γ*t expression. After the identification of Th17 cells as the kernel of severe asthma (Figures 3 and 4), we measured the expression of GATA3 and ROR*γ*t in animal and cellular models of severe asthma. (a) Lung WB detection of GATA3 and ROR*γ*t and relative protein expression in normal control, asthma, and severe asthma mice model. A significant level of ROR*γ*t expression was noted in severe asthma than in the other groups correlating with the higher Th17 cells expression (Figure 3) in severe asthma. (b) Lung WB detection of GATA3 and ROR*γ*t and relative protein expression in mice model with DHT, E2, and DHT/E2 influence. We observed a significant decrease in ROR*γ*t detection and expression after the addition of DHT in the severe asthma +DHT group as compared to severe asthma, severe asthma+E2, and severe asthma+ DHT/E2 group. (c) After the detection of BECs and measurement of IL-4 and IL-17 (Figures 4(a) and 4(b)), WB detection of GATA3 and ROR*γ*t and relative protein expression was performed in the BECs model. Th17 cells predominant BECs severe asthma model was induced by treating BECs with 100 *μ*g/ml HDM and 100 ng/ml LPS, 100 *μ*g/ml HDM to induce T2 asthma (asthma), and PBS for normal controls. Severe asthma was mediated by a significant higher level of ROR*γ*t expression than the normal control and asthma group. (d) Androgen in severe asthma+ DHT decreased the ROR*γ*t detection but significantly higher ROR*γ*t detected in severe asthma+ E2 group and the ROR*γ*t detection in severe asthma+ DHT/E2 was in between the independent effect of DHT and E2 alone. The relative protein expression results were similar to WB results (^∗^*p* < 0.05, ^∗∗^*p* < 0.01).

After the identification of Th17 cells, we conducted WB analysis to detect ROR*γ*t and GATA3 in the animal and cellular severe asthma model. We detected significantly higher ROR*γ*t expression in severe asthma than in asthma and the normal control group (Figure 5(a)) correlating with the higher Th17 cells expression in severe asthma (Figure 3(a)). The severe asthma+E2 group increased the ROR*γ*t expression, and the addition of DHT (severe asthma+ DHT) significantly reduced ROR*γ*t expression than in severe asthma and severe asthma+E2 group (Figure 5(b)). Severe asthma+ DHT/E2 did not decrease ROR*γ*t to the level of severe asthma+ DHT. Similarly, the severe asthma group in the cellular model also showed significantly higher ROR*γ*t expression than the asthma and the normal control group (Figure 5(c)). Relative protein expressions were also shown in the figure and corresponded with WB results. Androgen in severe asthma+ DHT decreased the ROR*γ*t detection and significantly higher ROR*γ*t detected in severe asthma+ E2 group, and the ROR*γ*t detection in severe asthma+ DHT/E2 was in between the independent effect of DHT and E2 alone. The relative protein expression results were similar to WB results (Figure 5(d)).

### 3.6. Effects of DHT, E2, and DHT/E2 on MBD2 Detection in Animal and BECs Severe Asthma Model

**Figure 6:**
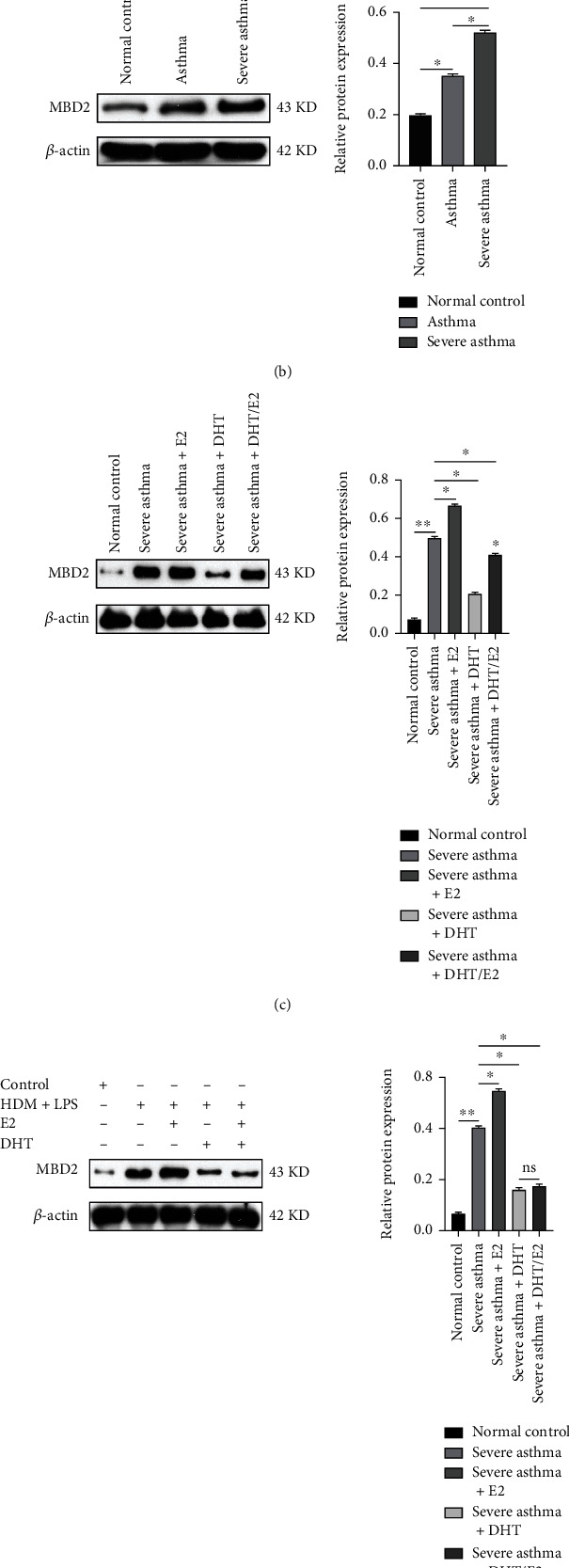
Effects of DHT, E2, and DHT/E2 on MBD2 detection in animal and BECs severe asthma model. (a) Immunohistochemistry analysis (anti-MBD2) of lung tissues from the severe asthma group showed more cells with stained MBD2 positive than asthma and normal control group, whereas the severe asthma+E2 group showed an elevated level of MBD2 positive and on the other hand severe asthma+ DHT reduced the MBD2 stained positive cells showing the androgen therapeutic possibility in MBD2-mediated severe asthma. (b, c) Western blot detection of MBD2 in different groups from the lung sample showed significantly higher MBD2 protein expression in severe asthma than in asthma and normal control group as shown in (b). Severe asthma+E2 showed an obvious increase in MBD2 than the severe asthma+ DHT, and DHT added group significantly reduced MBD2 expression than severe asthma and other hormone added group as shown in (c). Relative protein expression including DHT, E2, and DHT/E2 added groups are also shown in the figure. (d) Western blot detection of MBD2 from different groups of BECs asthma model including the DHT, E2, and DHT/E2 groups. Detected BECs were stimulated with 100 *μ*g/ml HDM as asthma, 100 *μ*g/ml HDM+100 ng/ml LPS as severe asthma, or PBS as a normal control group, sex hormones added in the designated group, and WB detected MBD2. The severe asthma+ DHT group significantly reduced MBD2 expression to the severe asthma and other hormone added group. Relative MBD2 protein expressions were also shown. Scale bar, 20 *μ*m. (^∗^*p* < 0.05, ^∗∗^*p* < 0.01, ns: not significant).

Lung histological analysis from the severe asthma group showed noticeably enhanced cells stained with MBD2 compared with the asthma group (Figure 6(a)). The addition of the E2 in severe asthma+E2 group extensively increased the MBD2 stained cells, while the severe asthma+ DHT group markedly reduced MBD2 (Figure 6(a)). The severe asthma+ DHT/E2 group did not reduce MBD2 to the level of severe asthma +DHT. WB detected MBD2 protein expression in the animal model, and significantly higher MBD2 expression was observed in severe asthma (Figure 6(b)). The severe asthma+E2 group was associated with higher MBD2 expression, while the severe asthma+ DHT group greatly reduced the MBD2 (Figure 6(c)). Severe asthma+ DHT/E2 did not reduce MBD2 to the level of severe asthma+ DHT (Figure 6(c)). Similarly, WB detection of MBD2 in the BECs model and under the DHT, E2, and DHT/E2 effect also showed the same result as above (Figure 6(d)).

### 3.7. Effects of DHT, E2, and DHT/E2 on MBD2 Detection and Expression under MBD2 Silencing or Overexpression (OE) in BECs Severe Asthma Model

**Figure 7:**
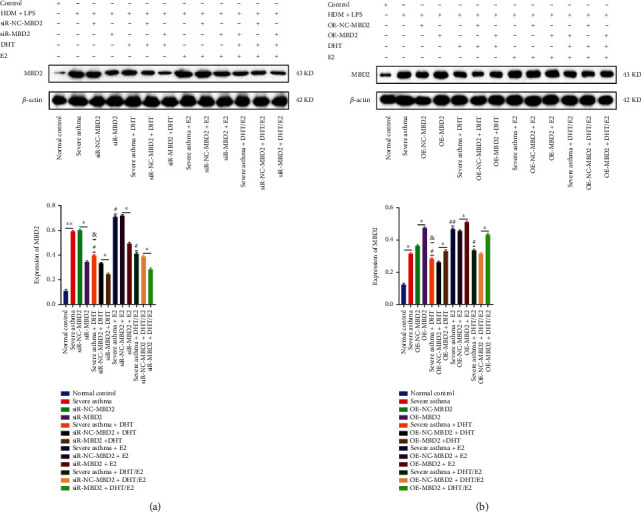
Effects of DHT, E2, and DHT/E2 on MBD2 detection and expression under MBD2 silencing or overexpression (OE) in BECs severe asthma model. (a) The expression of MBD2 at its silencing under the influence of DHT, E2, and DHT/E2 has been shown. Severe asthma was mediated by increased MBD2 expression, and as obvious, SiR-MBD2 reduced the MBD2 which was cross-checked by SiR-NC-MBD2. Severe asthma + DHT reduced the expression of MBD2 as compared with severe asthma, severe asthma +E2, and severe asthma +DHT/E2 group showing the androgenic therapeutic potential. Similarly, reduced MBD2 detection was noted with silencing in severe asthma +DHT (SiR-MBD2+DHT) as compared with SiR-MBD2 in severe asthma. Severe asthma + E2 was associated with increased MBD2 as compared with the severe asthma group and silencing of MBD2 decreased its detection, while SiR-NC-MBD2 was associated with significantly increased MBD2 expression. SiR-MBD2+E2 showed comparatively higher MBD2 detection than the SiR-MBD2+DHT. The severe asthma+ DHT/E2 group was observed with reduced MBD2 detection better than severe asthma+ E2, but not to that level of severe asthma+ DHT. Expression of MBD2 protein at its silencing also has been shown and corresponded with WB results. (b) The expression of MBD2 at its OE under the influence of DHT, E2, and DHT/E2. Severe asthma was associated with increased MBD2 detection than the normal control, and OE-MBD2 increased its detection, while as usual, it was counter-checked by OE-NC-MBD2. Androgen in (severe asthma + DHT) reduced the MBD2 detection, and the OE-MBD2+DHT group increased the MBD2 detection, but the detection was lowest than all the OE groups showing the androgenic therapeutic potential. Androgen estrogen ratio in severe asthma+ DHT/E2 did not reduce the MBD2 expression as severe asthma+ DHT. The expression of MBD2 protein at its OE was also shown in the figure. #Severe asthma vs severe asthma+ DHT, severe asthma+E2, and severe asthma+ DHT/E2. ##Severe asthma vs severe asthma+E2 and & severe asthma+ DHT vs severe asthma+ DHT/E2 (^∗^*p* < 0.05, ^∗∗^*p* < 0.01, #*p* < 0.05, ##*p* < 0.01, &*p* < 0.05).

We conducted WB analysis to detect MBD2 under its silencing and overexpression in a cellular severe asthma model and under the influence of DHT, E2, and DHT/E2. Severe asthma was associated with increased MBD2 expression and confirmed by its silencing and OE (Figures 7(a) and 7(b)). MBD2 detection in the severe asthma+ DHT group was lower than severe asthma, severe asthma +E2, and severe asthma+ DHT/E2 (Figures 7(a) and 7(b)). Severe asthma + DHT group showed lower MBD2 detection and expression during OE as compared with OE in the severe asthma+ E2 and severe asthma+ DHT/E2 group (Figure 7(b)). We noted comparatively higher MBD2 detection and expression in the severe asthma+ E2 group during its silencing than the silencing in severe asthma+ DHT and severe asthma+ DHT/E2 group (Figure 7(a)). The expression of MBD2 protein at its silencing and OE coincided with the WB detection results.

### 3.8. Effects of DHT, E2, and DHT/E2 on Differentiation and Expression of Th2/Th17 cells (IL-4/IL-17) under MBD2 Silencing or OE in BECs Severe Asthma Model

**Figure 8:**
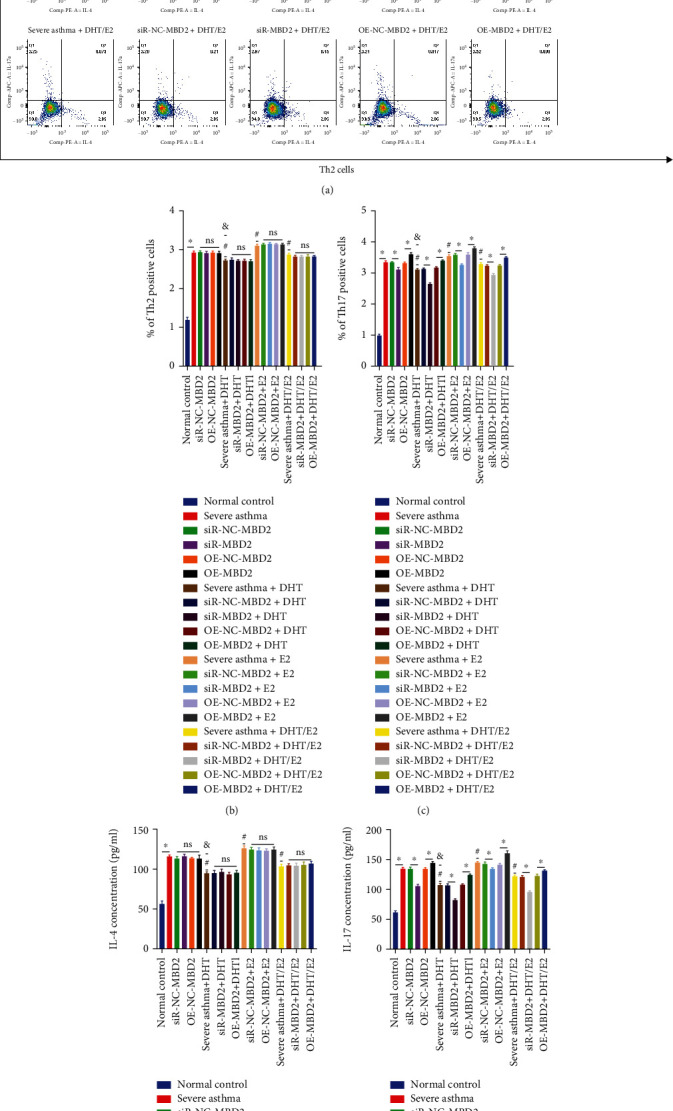
Effects of DHT, E2, and DHT/E2 on differentiation and expression of Th2/Th17 cells (IL4/IL17) under MBD2 silencing or OE in BECs severe asthma model. After the transfection of BECs, the addition of HDM, LPS, DHT, E2, and DHT/E2 in the designated groups and coculture with CD4+ T cells, flow cytometric analysis was performed to detect Th2/Th17 cells and ELISA for IL-4/IL-17 concentration from culture supernatant. (a) Th2/Th17 cells detection under silencing and OE of MBD2 and with DHT, E2, and DHT/E2 influence. Severe asthma was mediated by Th17 cells. OE-MBD2 was associated with an increase in Th17 cells, while silencing decreased Th17 cells almost below the severe asthma level. The addition of androgen in severe asthma (severe asthma+ DHT) decreased the differentiation of the Th17 cells, and OE-MBD2 was associated with increased Th17 cells differentiation, while MBD2 silencing automatically reduced the differentiation at a much lower level as compared with silencing without DHT in severe asthma showing Th17 cells differentiation was MBD2 mediated and DHT decreased Th17 cells differentiation of severe asthma via regulating MBD2. Estrogen was associated with higher Th17 cells differentiation in severe asthma+E2 and MBD2-OE+E2 resulted in much higher Th17 cells differentiation as compared to OE in severe asthma without E2 (OE-MBD2), while the silencing, as usual, was associated with decreased differentiation of Th17 cells just near or below the severe asthma level showing the potential role of E2 in severe asthma. The addition of DHT/E2 in severe asthma+ DHT/E2 decreased Th17 cells, but the decrease was not to that level of DHT alone. Thus, androgen (severe asthma+ DHT) alone showed the most potent effect in decreasing Th17 cells in severe asthma via MBD2. (b, c) % of Th2 and Th17 positive cells under MBD2 silencing or OE with the influence of DHT, E2, and DHT/E2 in different groups of severe asthma. (d) ELISA measurement of IL-4 and IL-17 concentration under MBD2 silencing or OE with the influence of DHT, E2, and DHT/E2 in different groups of severe asthma also correlated with flow cytometry results. #Severe asthma vs severe asthma+ DHT, severe asthma+E2, and severe asthma+ DHT/E2 and & severe asthma+ DHT vs severe asthma+ DHT/E2. (^∗^*p* < 0.05, #*p* < 0.05, &*p* < 0.05; ns: not significant).

We conducted flow cytometry analysis to evaluate the effect on Th2/Th17 cells differentiation under MBD2 silencing or OE together with hormonal effect. Severe asthma was mediated by Th17 cells and MBD2-OE increased Th17 cells differentiation, while silencing showed the opposite results. We did not see significant changes in Th2 cells differentiation during MBD2-OE, and silencing though silencing was associated with slightly higher Th2 cells than severe asthma (Figure 8(a)). DHT reduced the Th17 cells, while as usual MBD2-OE in severe asthma+ DHT group increased Th17 cells, and silencing was associated with decreased cells (Figure 8(a)). Estrogen (E2) increased the Th17 cells differentiation in severe asthma+E2 group, and OE and silencing of MBD2 were associated with increased and decreased level of Th17 cells, respectively. However, OE-MBD2+E2 was associated with higher Th17 cells differentiation than the OE-MBD2 of severe asthma showing Th17 cells differentiation was MBD2 dependent (Figure 8(a)). The addition of DHT/E2 (severe asthma+ DHT/E2) decreased Th17 cells, but the decrease was not to that level of severe asthma+ DHT group (Figure 8(a)). The percentage of Th2/Th17 cells under MBD2 silencing or OE with hormonal influence coincided with the flow cytometry results (Figures 8(b) and 8(c)). Concentration of IL-4/IL-17 from culture supernatant also coincided with (Figures 8(a), 8(b), 8(c), and 8(d)) results.

### 3.9. Effects of DHT, E2, and DHT/E2 on Detection and Expression of ROR*γ*t and GATA3 under MBD2 Silencing or OE in BECs Severe Asthma Model

**Figure 9:**
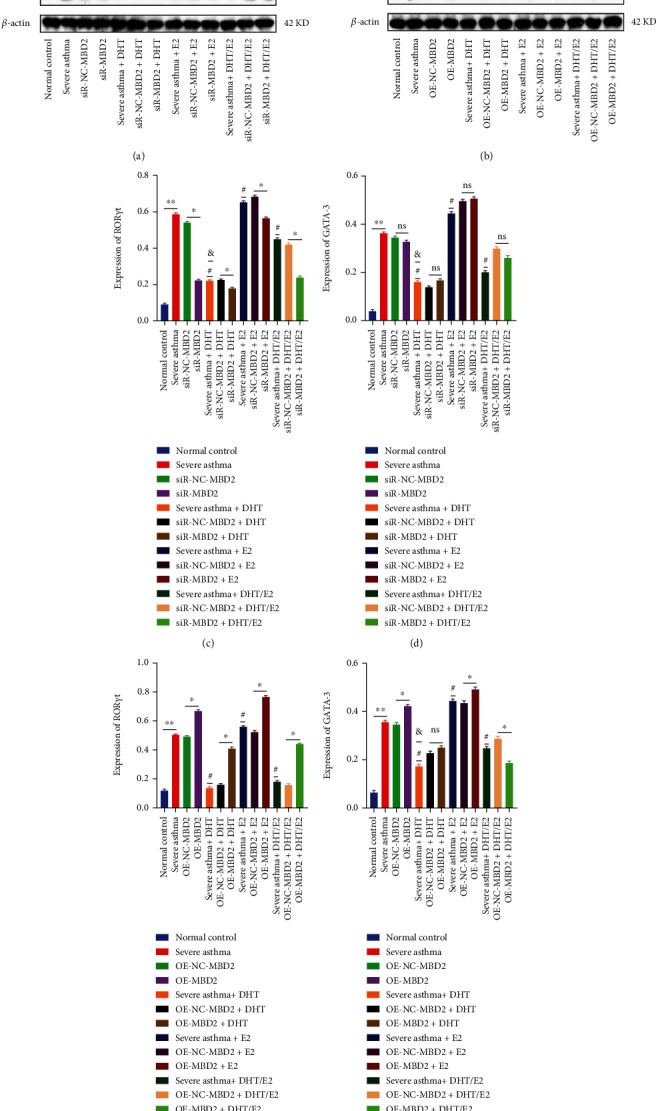
Effects of DHT, E2, and DHT/E2 on detection and expression of ROR*γ*t and GATA3 under MBD2 silencing or OE in BECs severe asthma model. (a) We detected the expression of GATA3 and ROR*γ*t at MBD2 silencing under the influence of DHT, E2, and DHT/E2 by WB. Comparatively, higher ROR*γ*t is detected than the GATA 3 in the severe asthma group coinciding with the higher Th17 (IL-17) cells as shown in Figures 8(a), 8(c), and 8(d). Silencing of MBD2 decreased the detection of ROR*γ*t in severe asthma significantly, but not GATA3 which was counter confirmed by SiR-NC-MBD2. The addition of androgen in the severe asthma+ DHT group significantly reduced ROR*γ*t detection than the GATA3 as compared with severe asthma. SiR-NC-MBD2 and SiR-MBD2 group of DHT showed significant changes in ROR*γ*t detection, but not GATA3 showing androgenic potential in decreasing ROR*γ*t via MBD2. Estrogen in severe asthma+E2 significantly increased the ROR*γ*t detection, while silencing of MBD2 decreased the ROR*γ*t detection, but not GATA3. Androgen estrogen in severe asthma+ DHT/E2 decreased ROR*γ*t detection than severe asthma and severe asthma+E2, but not to the level of alone as in severe asthma+ DHT group showing the most potent effect of DHT in ROR*γ*t detection regulating MBD2. (b) WB detection of GATA3 and ROR*γ*t at MBD2 OE under the influence of DHT, E2, and DHT/E2. Severe asthma was mediated by higher ROR*γ*t, and OE-MBD2 persistently showed higher ROR*γ*t detection as confirmed by OE-NC-MBD2. OE-MBD2 in the androgen group (OE-MBD2+ DHT) increased ROR*γ*t detection; however, the detection was lower than the OE in severe asthma (OE-MBD2) showing androgenic potential, and as expected, OE-NC-MBD2+ DHT decreased the detection. Interestingly, OE-MBD2+DHT and OE-NC-MBD2+DHT did not significantly change the detection of GATA3 which also showed the potential role of DHT on ROR*γ*t expression. OE-MBD2 in the estrogen group (OE-MBD2+E2) was associated with a significant increase in ROR*γ*t than without OE in severe asthma+E2. Expression of ROR*γ*t and GATA3 protein under MBD2 silencing or OE with the effect of DHT, E2, and DHT/E2 correlated with WB detection (c, d, e, f). #Severe asthma vs severe asthma+ DHT, severe asthma+E2, and severe asthma+ DHT/E2 and & severe asthma+ DHT vs severe asthma+ DHT/E2. (^∗^*p* < 0.05, #*p* < 0.05, ##*p* < 0.01, &*p* < 0.05, and ns: not significant).

A comparatively higher ROR*γ*t expression was detected than the GATA 3 (Figure 9(a)) coinciding with the higher Th17 (IL-17) cells expression in severe asthma as shown in Figures 8(a)–8(c). Silencing of MBD2 decreased the detection of ROR*γ*t in severe asthma significantly but not GATA3 which was counter confirmed by SiR-NC-MBD2. OE of MBD2 was associated with increased ROR*γ*t detection (Figure 9(b)). DHT decreased the ROR*γ*t detection, and silencing/OE of MBD2 decreased/increased ROR*γ*t, respectively, with DHT influence (Figures 9(a) and 9(b)). However, silencing/OE of MBD2 in severe asthma+ DHT group did not significantly change the GATA3 detection within the group (Figures 9(a) and 9(b)). Higher ROR*γ*t and GATA3 detection noted with E2 (severe asthgma+E2) as compared to severe asthma (Figures 9(a) and 9(b)). Silencing/OE of MBD2 in this group decreased/increased ROR*γ*t detection, but the detection of GATA3 was not significant with silencing of MBD2 (Figures 9(a) and 9(b)). Severe asthma+ DHT/E2 group showed intermediate level of ROR*γ*t detection as compared with the severe asthma+ DHT and severe asthma+E2 group. SiR-MBD2 and SiR-NC-MBD2 with DHT/E2 did not show significant changes on GATA3 detection (Figures 9(a) and 9(b)). Expression of ROR*γ*t and GATA3 with MBD2 silencing under the effect of DHT, E2, and DHT/E2 corresponded with WB detection (Figures 9(c) and 9(d)). Expression of ROR*γ*t and GATA3 with MBD2 OE under the effect of DHT, E2, and DHT/E2 corresponded with WB detection (Figures 9(e) and 9(f)).

## 4. Discussion

### 4.1. Major Findings

In the present study, we found that DHT attenuated BECs regulated Th17 cells (IL-17) differentiation and expression via regulating MBD2 in Th17 cells predominant neutrophilic severe asthma. Increased detection and expression of MBD2, Th17 cells (IL-17), and ROR*γ*t were noted in severe asthma, and DHT decreased the detection and expression of all. Th17 cells differentiation was MBD2 dependent, and DHT attenuated the differentiation and expression of Th17 cells (IL-17) via regulating MBD2. BECs served as the potential therapeutic target of DHT in cellular level.

### 4.2. Importance of this Study

Broadly speaking, we observe the two peaks of asthma in human life. The first peak is childhood asthma, and the second is adult-onset/old age asthma [43]. The second peak, non-T2 asthma (T2 low) with Th17 cells and neutrophil predominance, is resistant or less responsive to ICS and severe in nature [1, 2]. Surprisingly, severe asthma accounts for only about 5-10% of the total asthmatic population but does carry a heavy burden with high mortality, morbidity, and health expenses [44]. Currently, we are in immense need of a new therapeutic agent that not only treats severe asthma but also overcomes therapy-resistant issues and reduces mortality and morbidity.

#### 4.2.1. Establishment of hypothesis

Asthma is more prevalent in men than in women with aging as the androgen level decreases [25, 26]. The aggravation of asthma symptoms noted during the premenstrual and menstrual phase seems to be correlated with reduced testosterone level [27], showing that androgens might potentially be a therapeutic option. Recently, we reviewed the correlative association of Th17 cells, MBD2 expression, and sex hormones fluctuation status [19]. Therefore, we hypothesized that androgen has a potential therapeutic role in MBD2-mediated Th17 cells predominant neutrophilic severe asthma.

#### 4.2.2. Clinical Study

We enrolled patients in a normal control group, an asthma group, and a severe asthma group to evaluate the sex hormonal fluctuation status with asthma outcome by measuring the concentration of the DHT, the E2, and the DHT/E2 ratio. We found that severe asthma was associated with a comparatively higher E2 and a lower DHT level. These results and our previous study [16] together showed the increased level of association of E2 and MBD2 in Th17 cells predominant severe asthma, and the inverse association with DHT shows the possibility of reduced level of androgen as a causative mechanism for asthma pathogenesis which also shows the therapeutic potential of androgen.

#### 4.2.3. Animal Model and BECs Model of Severe Asthma

In order to better understand the severe asthma phenomenon and sex hormonal therapeutic potential, we constructed Th17 cells mediated neutrophil-predominant severe asthma animal model [32, 33] and BECs model [40] based on our previous study and continued further. We observed variations in doses of subcutaneous injection of androgen and estrogen in experimental mice in other field of study. However, we still do not have published therapeutic doses in severe asthma of animal and cellular model. Based on our preliminary evaluation on androgen estrogen doses and other studies, we injected 3.6 *μ*g/g body wt. of DHT [34, 45], 2.5 *μ*g/mice of E2 [35, 45], 3.6 *μ*g/g body wt. of DHT:2.5 *μ*g of DHT/E2 in the allocated groups of experimental mice, and 10 nM of DHT in BECs severe asthma model [41, 42]. However, we need more extensive and elaborative studies on various multiple doses of androgen. The severe asthma animal model was confirmed with the measurement of Mch induced AHR, BALF cells count (total, NEU, and EOS), lung histopathological analysis and relative protein expression, and Th17 cells (IL-17) differentiation, and all the parameters except BALF eosinophils were significantly increased in severe asthma showing the successful establishment of severe asthma model.

### 4.3. Increased Detection and Expression of MBD2, Th17 Cells (IL17), and ROR*γ*t in Severe Asthma

We detected higher expression of MBD2 in severe asthma, and study has shown its involvement in severe asthma pathogenesis [13, 14]. Spleen is well-known as the classical observational target for immune cells, and we noted an abundance of Th17 cells with a higher proportion of Th17 cells from splenocytes in severe asthma and was correlated with higher IL-17 protein concentration, and the same result was also seen in BECs model. Similarly, WB detected higher ROR*γ*t in severe asthma corresponding with Th17 (IL-17) cells in both models. Thus, the increased expression of MBD2, Th17 cells (IL17), and ROR*γ*t in severe asthma was perceived.

### 4.4. DHT Decreased the MBD2, Th17(IL-17) Cells, and ROR*γ*t Detection and Expression

The effect of DHT and E2 on MBD2, Th17 cells (IL17), and ROR*γ*t was also explored in animal and cellular model of asthma. DHT decreased all the markers of severe asthma (MBD2, Th17cells (IL-17), and ROR*γ*t) showing therapeutic potential, while E2 increased all the markers as it aggravated severe asthma. The effect of the DHT/E2 ratio was in between the independent effect of DHT and E2, and we did not consider it as a therapeutic potential. Collectively, androgen decreased the markers of severe asthma showing the therapeutic potential.

### 4.5. Th17 Cells (IL-17) Differentiation was MBD2 Dependent

In our previous animal model study [13, 14], significantly increased MBD2 expression was observed from splenocytes, and spleen corresponds to the traditional observation target of immune cells. The lung is one of the effector organs of immunity, and the higher expression of MBD2 was the hallmark in severe asthmatic lung. We performed in vitro splenocyte tests to determine if MBD2 is involved in the development of severe asthma and discovered that IL-17 protein expression dramatically increased along with MBD2 overexpression and decreased with MBD2 silencing. The MBD2 gene's overexpression or silencing coincided with a consistent change in the proportion of Th17 cells. Meanwhile, MBD2 KO mice were associated with comparatively less asthma symptoms, and interestingly, MBD2 was completely abolished in MBD2 KO mice. These experiments demonstrate that MBD2 is involved in severe asthma via influencing Th17 cell development and IL-17 release.

Similarly, in the BECs severe asthma model of our study, the correlative association between Th17 cells and MBD2 expression was also explored. We noted OE of MBD2 increased the differentiation of the Th17 cells, and silencing resulted in decreased differentiation. At the same time, IL-17 protein expression increased significantly with OE of MBD2 and decreased with silencing showing the involvement of MBD2 in severe asthma by affecting Th17 cells differentiation and IL-17 secretion.

### 4.6. DHT Decreased Th17 Cells (IL17) Differentiation via Regulating MBD2

To ascertain the working mechanism of how DHT was involved in decreasing the Th17 cells differentiation (IL-17) either by directly affecting it or via MBD2, we further evaluated the role of DHT on MBD2, Th17 cells (IL-17), and ROR*γ*t expression under MBD2 silencing or OE.

First, when we evaluated DHT effect on MBD2 detection under MBD2 silencing or OE, the detection and expression level of MBD2 correlated with its silencing or OE in each group and comparing the MBD2 expression of different groups (severe asthma, DHT, E2, and DHT/E2) confirmed the potent role of DHT in MBD2 expression. Thus, silencing or OE of MBD2 corresponds with the MBD2 detection in the respective groups, and DHT decreases MBD2 expression in severe asthma.

Second, we analyzed the DHT effect on Th17 cells (IL-17) differentiation under MBD2 silencing or OE. Severe asthma was mediated by increased concentration of Th17 cells, and as usual, E2 increased Th17 cells of severe asthma, while DHT administration decreased it, showing the therapeutic potential, while the combined effect of DHT/E2 did not show the welcoming effect on Th17 cells and was not a therapeutic potential. We noticed the significant changes on % of Th17 cells (IL-17), but not on % of Th2 cells (IL-4) in severe asthma. Thus, correlative analysis and comparison of Th2/Th17 (IL-4/IL-17) differentiation under MBD2 OE or silencing with DHT, E2, and DHT/E2 effect showed the potential role of DHT in Th17 cells (IL-17) downregulation.

Third, we also evaluated the expression of GATA3/ROR*γ*t under the same conditions, and the detection and expression of GATA3/ROR*γ*t were similar to the Th2/Th17 cells (IL-4/IL-17) results as above. However, OE of MBD2 in E2 and DHT/E2 added groups increased GATA3 expression showing the potential role of DHT mainly in ROR*γ*t regulation. Thus, DHT action on downregulation and expression of ROR*γ*t with silencing or OE of MBD2 were explored and clarified.

### 4.7. BECs Served as a Cellular Target of DHT in Severe Asthma

BECs can directly regulate Th2/Th17 cells differentiation [40], and all the result of Section 4.6 showed BECs as a potential cellular therapeutic target of DHT in Th17 cells predominant severe asthma.

### 4.8. Future Directions

This study is the first to evaluate the androgen therapeutic potential in severe asthma Th17 cells regulation via MBD2. That is why, we have constructed a highway and a long way to go on this for exploration of more mysteries of severe asthma. In addition, in vivo extensive animal and human studies should be carried out in the future to clearly understand the androgenic therapeutic potential in order to evaluate the mechanism of action and how the mechanism differs from the working mechanism from the ICS treatment in severe asthma. We also need more extensive and elaborative studies on various multiple doses of androgen. We further expect linking of epigenetics and its components with androgenic therapy to further evaluate how the genetic and environmental factors of epigenetics modulate asthma severity and how understanding this complexity is beneficial for asthma pathobiology and generating a hormonal-based patient approach therapy.

## 5. Conclusions

In conclusion, androgen, as a novel hormonal therapeutic agent, attenuates the BECs regulated Th17 cells (IL-17) differentiation and expression via MBD2 in Th17 cells predominant neutrophilic severe asthma showing new hope for therapeutic prospects and overcoming therapy-resistant issues of severe asthma.

## Figures and Tables

**Table 1:** Clinical characteristics of participants.

Items	HCs (*n* = 19)	Asthma (*n* = 29)	Severe asthma (*n* = 29)	*p* value
Age (y), M ± SD	51.73 ± 6.64	57.82 ± 10.51	62.31 ± 10.14	0.002
Sex M/F, n/n (%/%)	9/10 (47.4/52.6)	11/18 (37.9/62.1)	26/3 (89.7/10.3)	<0.001
BMI (kg/m^2^), M ± SD	23.99 ± 2.68	25.23 ± 3.95	22.54 ± 3.28	0.015
Smoking history, *n* (%)				0.024
Never-smoker	13 (68.4)	23 (79.3)	13 (44.8)	
Ex-smoker	6 (31.6)	6 (20.7)	16 (55.2)	
ACT, M ± SD		22.75 ± 1.29	12.93 ± 3.16	<0.001
Lung function indexes, median (IQR)				
FEV_1_ (L)	2.6 (2.4-3.3)	1.8 (1.6-2.0)	1.0 (0.8-1.2	<0.001
FEV_1_/FVC (%)	81.2 (77.6-82.4)	69.3 (57.8-74.2)	39.2 (30.0-50.7)	<0.001
FEV_1_%predicted (%)	101.8 (98.1-103.0)	74.8 (65.7-87.5)	32.8 (26.2-42.0)	<0.001
Biochemical indexes, median (IQR)				
IgE (mg/l)	58.6 (27.0-442.0)	104.0 (58.2-228.4)	205.8 (161.5-1214.5)	0.008
Blood eosinophils (×10^9^)	0.15 ± 0.12	0.20 ± 0.19	0.36 ± 0.19	<0.001
Blood neutrophils (×10^9^)	3.75 ± 0.86	4.33 ± 1.32	4.94 ± 1.75	0.020

Note: Chi-square test, one-way analysis of variance (ANOVA), or Kruskal-Wallis test for comparisons followed by Dunn's multiple comparisons test. *p* < 0.05 was considered statistically significant. Abbreviations: HC: healthy control; M: male; F: female; BMI: body mass index; ACT: asthma control test; FEV1: forced expiratory volume in 1 second; FVC: forced vital capacity; IgE: immune globulin E; M ± SD: mean ± standard deviation; IQR: interquartile range.

## Data Availability

The original contributions presented in the study are included in the article, and further inquiries can be directed to the corresponding author(s).

## Abbreviations

Al (OH)3: Aluminum hydroxide
BALF: Broncho-alveolar lavage fluids
BECs: Bronchial epithelial cells
DHT: Dihydrotestosterone
E2: 17*β*-estradiol
ELISA: Enzyme linked immunosorbent assay
EOS: Eosinophils
FEV1: Forced expiratory volume in one second
FVC: Forced vital capacity
HDM: House dust mites
ICS: Inhaled corticosteroids
LPS: Lipopolysaccharides
MBD2: Methyl-CpG-binding domain protein 2
Mch: Methacholine
NEU: Neutrophils
OVA: Ovalbumin
ROR*γ*t: Retinoic acid receptor-related orphan nuclear receptor gamma t
RL: Lung resistance
STAT3: Signal transducer and activator of transcription 3
TGF-*β*: Transforming growth factor *β*
WB: Western blot.

## Supplementary Materials

Supplementary Materials Supplementary Figure 1: The figure shows the graphical representation of the abstract. In the clinical study phase, the concentration and ratio of androgen and estrogen were measured and correlated with the severe asthma status. Then, an animal model and bronchial epithelial cells (BECs) model of severe asthma was established to evaluate the role of MBD2 in the differentiation and expression of Th17 cells (IL17), the therapeutic potential of sex hormones in severe asthma, and the effect of sex hormones in BECs regulated Th17 cells differentiation via MBD2 at the cellular level. Androgen attenuated the differentiation of BECs regulated Th17 cells via MBD2 showing BECs as a therapeutic target of androgen.
