# Supplementary figures and images for: Androgen Plays a Potential Novel Hormonal Therapeutic Role in Th17 Cells Predominant Neutrophilic Severe Asthma by Attenuating BECs Regulated Th17 Cells Differentiation via MBD2 Expression
Source: Oxid Med Cell Longev. 2022 Aug 25;2022:3096528. doi: 10.1155/2022/3096528 (PMC9436621; doi:10.1155/2022/3096528)

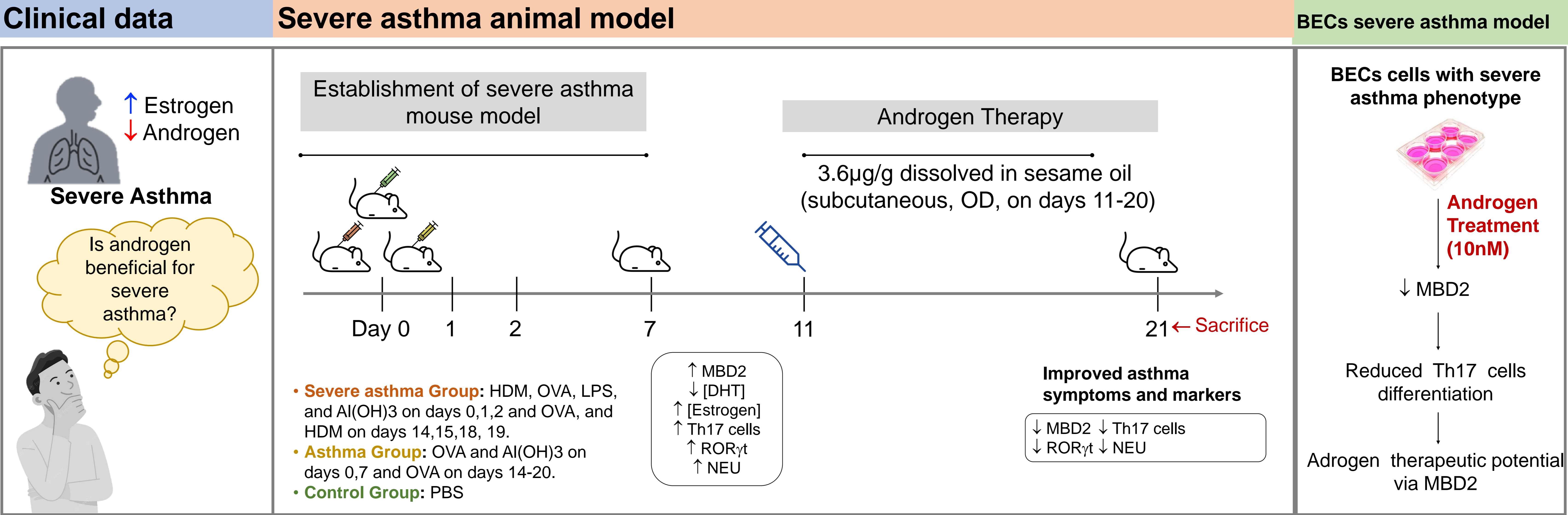

Supplement: Supplementary Materials — Supplementary Figure 1: The figure shows the graphical representation of the abstract. In the clinical study phase, the concentration and ratio of androgen and estrogen were measured and correlated with the severe asthma status. Then, an animal model and bronchial epithelial cells (BECs) model of severe asthma was established to evaluate the role of MBD2 in the differentiation and expression of Th17 cells (IL17), the therapeutic potential of sex hormones in severe asthma, and the effect of sex hormones in BECs regulated Th17 cells differentiation via MBD2 at the cellular level. Androgen attenuated the differentiation of BECs regulated Th17 cells via MBD2 showing BECs as a therapeutic target of androgen. [file 3096528.f1.pdf]
